# Randomized, crossover, controlled trial on the modulation of cardiac coronary sinus hemodynamics to develop a new treatment for microvascular disease: Protocol of the MACCUS trial

**DOI:** 10.3389/fcvm.2023.1133014

**Published:** 2023-02-16

**Authors:** Helen Ullrich, Maximilian Olschewski, Thomas Münzel, Tommaso Gori

**Affiliations:** ^1^Department of Cardiology, Cardiology I, University Medical Center Mainz, Mainz, Germany; ^2^German Centre for Cardiovascular Research, Standort RheinMain, Mainz, Germany

**Keywords:** coronary microvascular dysfunction, microvascular angina, arterial microcirculation, coronary sinus, fractional flow reserve

## Abstract

**Background:**

Microvascular angina (MVA) is a frequent condition for which our understanding of the disease pathophysiology and therapeutic perspectives remain unsatisfactory. The current study is designed to test whether an improvement in microvascular resistances could be achieved by elevating backward pressure in the coronary venous system, based on the hypothesis that an increase in hydrostatic pressure could cause a dilatation of the myocardial arterioles, resulting in a reduction of vascular resistances. This approach might have potential clinical implications, as it might suggest that interventions aimed at increasing coronary sinus (CS) pressure might result in a decrease in angina in this subset of patients. The aim of our single-center, sham-controlled, crossover randomized trial is to investigate the effect of an acute increase in CS pressure on a number of parameters of coronary physiology, including parameters of coronary microvascular resistance and conductance.

**Methods and analysis:**

A total of 20 consecutive patients with angina pectoris and coronary microvascular dysfunction (CMD) will be enrolled in the study. Hemodynamic parameters including aortic and distal coronary pressure, CS and right atrial pressure, and the coronary microvascular resistance index will be measured at rest and during hyperemia in a randomized crossover design during incomplete balloon occlusion (“balloon”) and with the deflated balloon in the right atrium (“sham”). The primary end point of the study is the change in index of microvascular resistances (IMR) after acute modulation of CS pressure, while key secondary end points include changes in the other parameters.

**Discussion:**

The aim of the study is to investigate whether occlusion of the CS is associated with a decrease in IMR. The results will provide mechanistic evidence for the development of a treatment for patients with MVA.

**Clinical trial registration:**

https://clinicaltrials.gov/, identifier NCT05034224.

## Introduction

Microvascular angina pectoris (MVA) is a complex clinical condition in which functional and/or structural changes in the coronary microcirculation result in an increased vascular tone. MVA is diagnosed as symptoms of ischemia with objective evidence of impaired coronary microvascular function [e.g., an index of microvascular resistance (IMR) > 25] in the absence of obstructive epicardial disease (1, 2). While the clinical attention and academic interest in this field is growing, the mechanisms behind its pathophysiology and the therapeutic alternatives available remain incompletely investigated (3). Despite these uncertainties, there is a consensus that this condition is clinically relevant as it affects up to two-thirds of patients who suffer from stable angina and either have no epicardial coronary stenosis at angiography or have combined epicardial and microvascular disease (4).

Microvascular angina caused by a dysfunctional microvascular bed cannot be treated by standard revascularization therapies and the pharmacological strategies (e.g., nitrates, calcium channel blockers, ranolazine) available also provide limited benefit. As well, while pressure-controlled intermittent coronary sinus occlusion has been shown to improve microvascular resistances in the setting of acute myocardial infarction (5), there is little evidence that interventions may improve microvascular resistances in patients with chronic coronary syndromes.

As a possible alternative to pharmacological agents, the hypothesis of improving myocardial perfusion by diverting blood from the coronary venous system to an ischemic region (“venous retroperfusion”) has again gained attention during recent years. This therapy is based on the concept that an elevation in backward pressure in the coronary venous system provokes dilatation of the subendocardial arterioles, resulting in a relative reduction of vascular resistance in this area and a redistribution of blood flow to the ischemic subendocardial layers (6–8). Numerous studies confirm the efficacy of this approach for the therapy of patients who have angina and are not candidates for “traditional” revascularization (6). The study presented here aims to investigate the effect of an increase in coronary sinus pressure on microvascular function, a potentially new therapeutic concept.

## Materials and methods

### Overview

We investigate whether an increase in coronary sinus pressure leads to a change in coronary microvascular resistances in patients with angina pectoris due to microvascular dysfunction.

### Study design

The study is a sham-controlled, crossover, randomized trial to investigate the effect of changes in coronary venous pressure on microvascular resistances. The hypothesis of the study is that occlusion of the coronary sinus (CS) will be associated with a decrease in the index of microvascular resistances (IMR). The protocol complies with good clinical practice and the ethical principles described in the Declaration of Helsinki and has been approved by the local ethics committee. All patients will provide written informed consent before enrollment.

### Study population

Patients must meet all of the following inclusion criteria: chronic coronary syndrome (including patients with anginal equivalents); reversible ischemia on non-invasive testing; absence of epicardial stenosis that are compatible with the symptoms and the evidence of ischemia; evidence of microvascular dysfunction as demonstrated by an IMR ≥ 25; willingness to participate and ability to understand, read, and sign the informed consent; age > 18 years.

Patients will be excluded from eligibility for study enrollment if any of the following criteria applies: previous coronary artery bypass graft (CABG) with patent graft to the left anterior descending coronary (LAD) such that IMR cannot be measured; epicardial coronary disease (FFR < 0.80 with evidence of a focal stenosis) in the LAD territory; second and third degree atrioventricular block; severe valvular heart disease; any cardiomyopathy; pulmonary or renal disease; inability to provide informed consent; any disease reducing life expectancy. Patients unable to understand the scope of the study are classified as not able to give informed consent and are excluded for eligibility for study enrollment. Likewise, patients unable to consent, e.g., for a neurological damage, are treated as not able to give informed consent and are excluded for eligibility for study enrollment (Table 1).

**TABLE 1:** In- and exclusion criteria.

Inclusion criteria (ALL)	Exclusion criteria (ANY)
● Chronic coronary syndrome (including patients with anginal equivalents)	● Previous CABG with patent grafts such that IMR cannot be measured
● Reversible ischemia on non-invasive testing	● Epicardial coronary disease (FFR < 0.80 with evidence of a focal stenosis) in the LAD territory
● Evidence of microvascular disease with IMR = 25	● Second and third degree atrioventricular block
● Willingness to participate and ability to understand, read, and sign the informed consent	● Severe valvular heart disease
● Age>18 years	● Any cardiomyopathy
	● Pulmonary or renal disease
	● Inability to provide informed consent
	● Any disease reducing life expectancy

### Procedures

#### Recruitment

Patients will be enrolled among those treated at the Department of Cardiology, Cardiology I of the University Medical Centre Mainz or, eventually, cooperating centers.

#### Randomization

**FIGURE 1:**
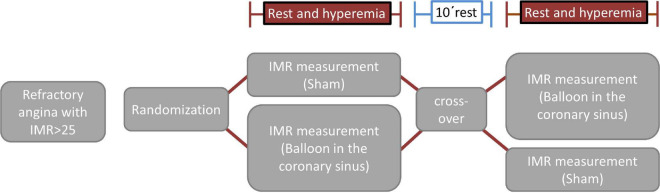
Randomization.

Microvascular function measurements will be made (both at rest and during hyperemia) twice in each patient in a sham-controlled, randomized, crossover design. Randomization will be performed after informed consent and if all inclusion criteria and no exclusion criteria are met. Patients will be randomized 1:1 to one of the two study arms (sham/balloon or balloon/sham) (Figure 1). After the first set of rest/hyperemia measurements in the first condition (balloon or sham, Figure 1), and a 10 min waiting time, patients will cross-over to the other condition (sham or balloon) and the measurements (rest/hyperemia) will be repeated.

For the measurements during balloon inflation, incomplete CS occlusion will be achieved with a Swan-Ganz catheter advanced into the CS with access through the femoral or jugular vein. The position of the balloon will be chosen in order to cause a ∼80–90% CS lumen reduction while maintaining stability throughout the measurements. For the sham measurements, the balloon will be kept deflated in the right atrium. Randomization will be done by a computer-generated random sequence (MedCalc, Ostend, Belgium).

#### Hemodynamic measurements

Coronary angiography and microvascular function assessments will be performed in the left anterior descending coronary artery using the wire-based thermodilution method. Five to seven thousand international units of intravenous heparin and intracoronary nitrates will be administered before measurements when systolic blood pressure is above 100 mmHg systolic. A guiding catheter will be advanced to the ostium of the left coronary. A pressure wire (Pressure Wire X; Abbott Vascular, Santa Clara, CA, United States) equipped with pressure sensor and thermistor will be advanced placed approximately 8 cm into the LAD after ostial equalization. Maximal hyperemia will be induced through intravenous adenosine (140 μg⋅kg^–1^⋅min^–1^) or, in patients with asthma, intracoronary papaverine (10 mg). The distal sensor of the pressure wire will be kept in the same position and the same hyperemic agent will be used for the two sets of measurements (sham and balloon). For the assessment of the mean transit time (Tmn) and IMR, 3 boli of 3 ml room-temperature saline will be injected briskly into the guiding catheter as per instructions for use. The presence of drift will be checked at the end of the measurements. All assessments will be performed using the CoroFlow software (Coroventis, Uppsala, Sweden).

### Trial endpoints

The primary analysis will be on the per-protocol principle (i.e., including all patients who are not protocol violators).

Primary endpoint: change in IMR during inflation of the CS balloon as compared to sham.

Key secondary endpoints: changes in the following physiological parameters

1. Aortic (Pa) and distal coronary pressure (Pd) at rest and during hyperemia (aortic and coronary pressure);
2. Pressure in the right atrium (Pra) and in the coronary sinus (Pcs);
3. RFR (resting flow ratio);
4. FFR calculated as Pd/Pa and as (Pd-Pcs)/(Pa-Pcs);
5. Tmn (transit mean time) at rest and during hyperemia, average of the three boli;
6. CFR: coronary flow reserve;
7. IMR calculated as [(*P*_*d*_ – CS) × *T*_*mn*_] and using the standard formula (IMRst = *P*_*d*_ × *T*_*mn*_). CFR is the ratio of baseline *T*_*mn*_/hyperaemic *T*_*mn*_.

### Safety

All measurements (including hemodynamic measurements) are applied as clinically indicated and according to CE certificate and the instruction for use. Vascular access is performed using the femoral or jugular vein; the expansion of the balloon is incomplete and performed using low pressure (2–4 ATM). The risk of trauma is thereby minimal. Thus, it is not to be expected that the participation in the study will lead to unneeded or harmful therapeutic interventions.

### Statistics

Statistical analysis will be performed with MedCalc (Ostend, Belgium).

#### Power calculation

The study is powered for the primary efficacy endpoint IMR. In the OxAMI-PICSO study (5), PICSO reduced the IMR in patients with no reflow after ST-elevation myocardial infarction from 45 [22–51] to 25 [19–36]. In unselected coronary artery disease patients, Mangiacapra et al. (9) reported a decrease in IMR from 27 ± 11 to 19 ± 9 after intracoronary enalaprilat; Suda et al. (10) reported a decrease in IMR from ∼28[21–35] to ∼19[17–23] (*P* < 0.0001, median decrease ∼35%) in response to fasudil. Luo (11) reported baseline IMR of 33 units in patients with microvascular dysfunction (i.e., our expected population), with a baseline SD of 7.6 units. When assuming a baseline IMR of 33 and conservatively using the pooled baseline SD of IMR 13 units of both treatment groups from Mangiacapra, and assuming a similar effect as that observed in the papers by Mangiacapra and Suda, this will result in an effect size of ∼0.69 (∼9/13). With this effect size and a power of 80%, 19 patients are needed. In case of missing data, this sample size could be increased to 25.

#### Statistical analysis

The primary analysis will be done for the IMR measurement. The primary hypothesis is H0: μExp = μSham, vs. H1: μExp ≠μSham, where μExp and μSham are the expected values of the IMR with experimental intervention (balloon) and sham. The primary analysis will be performed as within-subject comparison of the primary parameter. Statistical tests and effect estimates will be calculated using the Wilcoxon test. The effect of randomization and baseline measurements as covariates will be investigated. No treatment by time interaction is expected because the balloon from the experimental intervention will produce a transient increase in CS pressure which will normalize upon deflation. The primary comparison will be performed at a two-sided significance level of α = 0.05. Treatment differences will be displayed by adjusted estimates and 95% confidence intervals. Bias is minimized by randomized sequence allocation. The randomization ratio of the treatment sequences will be 1:1 without any stratification factors. Randomization with permuted blocks will be applied.

### Data management

Patient data will be pseudonymized and collected by the study team. Pseudonymized patient data will be stored digitally on an external hard-drive not connected to the clinical intranet and only accessible to the members of the study team. After 10 years of storage, data will be destroyed. It is not intended to give study participants’ data to a third party. All data will be analyzed after the last patient is discharged from index hospitalization. No interim analysis is intended. However, a Data Safety Monitoring Board consisting of two physicians not affiliated with the study will monitor the safety of the subjects throughout the study. In case a study participant withdraws consent after having his data collected from him, the patient’s data will be anonymized.

### Ethics and publication policy

The protocol has been approved by the local state medical association’s ethics committee. In accordance with the WMA Declaration of Helsinki–Ethical Principles for Medical Research Involving Human Subjects, researchers, authors, sponsors, editors, and publishers all have ethical obligations with regard to the publication and dissemination of the results of research. Researchers have a duty to make publicly available the results of their research on human subjects and are accountable for the completeness and accuracy of their reports. All parties should adhere to accepted guidelines for ethical reporting. Negative and inconclusive as well as positive results must be published or otherwise made publicly available. Sources of funding, institutional affiliations and conflicts of interest must be declared in the publication. Reports of research not in accordance with the principles of this Declaration should not be accepted for publication. The PI of this study, recognizing the seminal importance of this investigation, is committed to the unrestricted and widespread dissemination of all primary and secondary endpoint results and tertiary analyses. At the conclusion of the study, an abstract reporting the primary results will be prepared by the Principal Investigators and presented at an annual scientific meeting. A publication will similarly be prepared for publication in a reputable scientific journal. Following analysis and presentation of the primary endpoint results, active participation of all study group members will be solicited for data analysis and abstract and manuscript preparation and therefore included as co-authors. Submission of all abstracts and publications regarding the primary endpoint and secondary endpoints from the study requires approval by the Principal Investigators after review by all members of the study group.

### Insurance

Since all procedures (except for randomization) are clinically indicated and acknowledged in the current literature, a study insurance is not planned.

### Trial status

Data collection is ongoing.

### Financing

The study will be financed by own means of the Department of Cardiology of the University Medical Center Mainz (=Sponsor) and means of the W3-Professorship of Translational myocardial and cardiovascular function. Additionally, the research project will be supported by the Clinician Scientist program of Helen Ullrich, which was granted by the German Centre for Cardiovascular Research (DZHK).

## Discussion

To assess coronary circulatory function, three different physiological indexes were measured in the present study. FFR is defined as the ratio of maximal coronary blood flow in a diseased artery to maximal coronary blood flow in the same artery without stenosis. FFR is a surrogate marker of inducible myocardial ischemia caused by epicardial coronary stenosis (12). CFR is the ratio of hyperemic to baseline flow and is a marker of the integrity of both epicardial and microvascular domains of coronary circulation. Therefore, CFR represents the microvascular status when there is no significant epicardial disease. IMR is the minimum achievable coronary microcirculatory resistance and a more specific marker of the coronary microcirculation than CFR. It is calculated as the ratio of distal coronary pressure to coronary flow at hyperemia and presented in unit.

Approximately half of patients undergoing diagnostic coronary angiography for typical angina symptoms do not have significant obstructive coronary stenoses (10). This large number of patients rarely receive a definitive diagnosis, are often inappropriately labeled, and by and large remain symptomatic (13). Half of these patients suffer from CMD. The disease is associated with higher rates of serious cardiovascular events, making identification of CMD a therapeutic target with unmet needs (13). In this context, MVA still represents an underestimated clinical challenge, which, however, is increasingly proving to be an important factor in cardiac prognosis (14). In their prospective study, Suda et al. (10) showed that coronary functional abnormalities, including epicardial coronary spasm, decreased microvascular vasodilation, and increased microvascular resistance, frequently coexist in patients with angina and non-obstructive coronary artery disease. Here, the IMR correlated with the occurrence of severe cardiovascular events, and an IMR of 18.0 turned out to be the best cut-off value. Our randomized and sham-controlled trial investigates the acute effect of increasing coronary sinus pressure on microvascular resistance in patients with MVA. Our hypothesis states that occlusion of the CS is associated with a decrease in IMR. This retrograde treatment approach to myocardial ischemia through the coronary venous system has been studied in the past in the context of acute coronary syndrome, coronary revascularization, and cardiothoracic surgery (15). Several small studies support the approach that occlusion of the CS may preserve ischemic myocardium and improve microvascular perfusion and function (5, 12, 15, 16). In this context, the results of our work may provide evidence for a possible new therapeutic strategy.

Initial evidence from animal studies and a series of patients appears to support the hypothesis that coronary sinus occlusion (CSO) improves microvascular function: Ido et al. (15) demonstrated that CSO lead to dilatation of the subendocardial arterioles, resulting in a significant reduction of vascular resistance in this area and a redistribution of blood flow to these ischemic subendocardial layers. In a small case series by Giannini et al. (16), patients with microvascular angina showed a clinical improvement after CSO, an effect similar to that shown in the randomized, sham-controlled trial COSIRA (12) (improvement of 2 Canadian cardiovascular society angina classes in 35% of the patients). In the paper by de Maria et al. (5), percutaneous-intermittent CSO (PICSO) improved microvascular perfusion and resistances in patients with no reflow after ST-elevation myocardial infarction. Finally, we recently provided mechanistic evidence that CSO might improve microvascular function in a patient with evidence of severe CMD: in a recent publication (Gori T, Eurointervention online), we described a case of a patient with MVA that benefited from the implantation of the coronary sinus reducer device. This 61-year old patient with a history of insulin-dependent diabetes, hypertension, and multiple PCIs underwent implantation of the coronary sinus reducer for chronic angina refractory to maximal therapy. His symptoms included a CCS III angina leading to 4 hospitalizations in the 6 months before the sinus reducer implantation. In order to better understand the pathophysiology of his symptoms, a full hemodynamic assessment was performed. The fractional flow reserve (FFR) in the left anterior descending (LAD) was 0.87, witnessing the absence of epicardial disease. In contrast, the index of microvascular resistances (IMR) was 63, documenting increased microvascular resistances as a mechanism of his symptoms. Based on this evidence, and on compassionate grounds, a coronary sinus reducer was implanted. After implantation, the “trans-sinus” gradient was 3mmHg. FFR and resting full-cycle ratio (RFR) were unchanged (0.93 and 0.85), but IMR dropped to 37. Since implantation (currently 12 months follow-up at the time of this writing), the patient has never been admitted again to hospital and is symptom-free.

Although this is just preliminary data, they suggest that the redistribution of blood from the subepicardial to the subendocardial space might be associated with a drop in total microvascular resistances and therefore relief of symptoms.

Our study has several limitations. First, the present study is a mechanistic study conducted exclusively at one center. In addition, only a relatively small number of patients will be enrolled in each of the two study groups. Furthermore, we only studied the effect of a single occlusion of the CS on microvascular function. Repeated CSO as well as a permanent CSO could alter the effects of a single CSO (15). Larger studies with follow-up periods are needed to build on the results and to investigate the role of potential new therapeutic strategies.

## Author contributions

HU and TG wrote the first draft. All authors provided critical appraisal and corrections and approved the submitted version.
